# Identification of Prostate Cancer-Related Circular RNA Through Bioinformatics Analysis

**DOI:** 10.3389/fgene.2020.00892

**Published:** 2020-08-14

**Authors:** Yu-Peng Wu, Xiao-Dan Lin, Shao-Hao Chen, Zhi-Bin Ke, Fei Lin, Dong-Ning Chen, Xue-Yi Xue, Yong Wei, Qing-Shui Zheng, Yao-An Wen, Ning Xu

**Affiliations:** Department of Urology, The First Affiliated Hospital of Fujian Medical University, Fuzhou, China

**Keywords:** prostate cancer, circRNA, bioinformatics analysis, circRNA–microRNA–mRNA interaction axis, signaling pathway

## Abstract

**Background:**

Prostate cancer (PCa) is one of the most common malignant tumors worldwide. Accumulating evidence has suggested that circular RNAs (circRNAs) are involved in the development and progression of various cancers, and they show great potential as novel biomarkers. However, the underlying mechanisms and specific functions of most circRNAs in PCa remain unknown. Here, we aimed to identify circRNAs with potential roles in PCa from the PCa expression profile.

**Methods:**

We used data downloaded from the Gene Expression Omnibus to identify circRNAs that were differentially expressed between PCa samples and adjacent non-tumor samples. Relative expression levels of identified circRNAs were validated by quantitative real-time PCR. Micro (mi)RNA response elements were predicted by the CircInteractome database, and miRNA target genes were predicted by miRDB, miRTarBase, and TargetScan databases. Gene ontology (GO) enrichment analysis and pathway analysis revealed the potential biological and functional roles of these target genes. A circRNA–miRNA–mRNA interaction network was constructed by Cytoscape. The interaction between circRNAs and miRNAs in PCa was thoroughly reviewed in the PubMed. Finally, the mRNA expression of these genes was validated by the Cancer Genome Atlas (TCGA) and Gene Expression Profiling Interactive Analysis (GEPIA) databases. The expression of proteins encoded by these genes was further validated by the Human protein Atlas (HPA) database.

**Results:**

A total of 60 circRNAs that were differentially expressed between PCa and healthy samples were screened, of which 15 were annotated. Three circRNAs (hsa_circ_0024353, hsa_circ_0085494, hsa_circ_0031408) certified the criteria were studied. The results of quantitative real-time PCR demonstrated that the expression of hsa_circ_0024353 was significantly downregulated in PC-3 cells when compared with RWPE-1 cells, while the expression of hsa_circ_0031408 and hsa_circ_0085494 was significantly upregulated in PC-3 cells when compared with RWPE-1 cells. GO and Kyoto Encyclopedia of Genes and Genomes analyses found that target genes were mainly enriched in metabolic processes and pathways involving phosphoinositide 3-kinase-Akt signaling, hypoxia-inducible factor-1 signaling, p53 signaling, and the cell cycle. A total of 11 miRNA target genes showing differential expression between PCa and healthy samples were selected, and their mRNA and protein expression were validated by GEPIA and HPA databases, respectively. Of these, *PDE7B*, *DMRT2*, and *TGFBR3* were identified as potentially playing a role in PCa progression. Finally, three circRNA–miRNA–mRNA interaction axes were predicted by bioinformatics: hsa_circ_0024353–hsa-miR-940–PDE7B, hsa_circ_0024353–hsa-miR-1253–DMRT2, and hsa_circ_0085494–hsa-miR-330-3p–TGFBR3.

**Conclusion:**

This study identified three circRNA–miRNA–mRNA interaction axes that might provide novel insights into the potential mechanisms underlying PCa development.

## Introduction

Prostate cancer (PCa) is one of the most common cancers worldwide, and its incidence has gradually increased in many counties (Huang et al., 2018). For example, it has the seventh highest incidence and tenth highest mortality of all cancers in Chinese males (Zhang et al., 2017). Accumulating evidence suggests that at least 15% of PCa patients are diagnosed at an advanced stage (Cooperberg et al., 2010). Radical prostatectomy and androgen deprivation therapy (ADT) are the mainstay for patients with localized and advanced prostate cancer, respectively (Xue et al., 2018), while the standard therapy for metastatic PCa remains ADT. Nevertheless, metastatic PCa patients typically progress to the castration-resistant stage after 1 or 2 years of ADT (Josefsson et al., 2019). Therefore, as the means to cure and prevent PCa remain limited in the clinic, the identification of new biomarkers to monitor and intervene in PCa carcinogenesis is imperative.

Circular RNAs (circRNAs) are a class of endogenous non-coding RNAs that have covalent closed-loop structures with no 5′ cap or 3′ poly(A) tail. Their ring structure makes them more stable than mRNAs. They have many roles, including acting as micro (mi)RNA sponges (Thomson and Dinger, 2016), regulating alternative splicing (Feng J. et al., 2019), modulating parental genes (Li et al., 2015), sequestering proteins (Du et al., 2017), and functioning as scaffolds (Feng Z. et al., 2019). CircRNAs are proven essential elements in the initiation and progression of human disease, especially cancers (Wilusz and Sharp, 2013), and recent studies (Meng et al., 2017; Zhang et al., 2018; Verduci et al., 2019; Vo et al., 2019; Xie et al., 2019; Li S. et al., 2020) indicated that they function as potential biomarkers for cancer diagnosis and treatment.

In this study, we aimed to identify circRNAs that might be involved in PCa carcinogenesis by analyzing the expression profile of PCa. Data downloaded from the National Center of Biotechnology Information (NCBI) Gene Expression Omnibus (GEO) were used to identify novel circRNAs that were differentially expressed in PCa. Gene ontology (GO) enrichment analysis and pathway analysis revealed the potential function of miRNA target genes. Finally, mRNA expression levels of these genes were validated by the Cancer Genome Atlas (TCGA) and Gene Expression Profiling Interactive Analysis (GEPIA) databases. The protein expression of these genes was further validated by the Human Protein Atlas (HPA) database.

## Materials and Methods

### CircRNA Screening

Differentially expressed circRNAs were screened and identified using the GEO2R online tool from the NCBI GEO database GSE140927. |LogFC| ≥ 1 and *P* < 0.05 were set as hub circRNA screened criteria.

### Annotation of CircRNAs

We used circBase (Glažar et al., 2014) and circPrimer (Zhong et al., 2018) (version 1.2) software to annotate circRNAs of interest. The position, strand, genomic length, spliced length, and parental gene symbol were extracted from circBase. The composition of circRNA exons and introns was demonstrated by circPrimer. Coding sequences, untranslated regions, and start and stop codons were annotated by circPrimer.

### Cell Culture

PC-3 and the human prostatic epithelial cell line RWPE-1 were purchased from the American Type Culture Collection (Manassas, VA). PC-3 cells were cultured in F-12K medium. The medium was supplemented with 10% fetal bovine serum (Invitrogen) and 5% penicillin–streptomycin (Invitrogen). RWPE-1 cells were cultured in keratinocyte serum-free medium supplemented with 50 mg/ml bovine pituitary extract, 5 ng/ml human epidermal growth factor, and 1% penicillin–streptomycin.

### Quantitative Real-Time PCR (qRT-PCR)

Total RNA was isolated from cells and tissues using RNAiso Plus reagent (Takara, Kusatsu, Shiga, Japan). Reverse transcription (RT) was performed with random primers using the PrimeScript^TM^ RT reagent kit with gDNA Eraser (Takara) to detect the expression of hsa_circ_0024353, hsa_circ_0085494, and hsa_circ_0031408. Glyceraldehyde 3-phosphate dehydrogenase (*GAPDH*) was amplified as an internal control. The DNA extraction kit (TIANGEN, Beijing, China) was used to extract genomic DNA from cells and tissues. PCR reactions were performed using Thermo Scientific DreamTaq PCR Master Mix (2×) (Thermo Fisher Scientific, Waltham, MA, United States). The amount of circRNAs and mRNA was quantified after the RT step using SYBR Green qPCR Master Mix (Takara) according to the manufacturer’s instructions. The primers used in this study were: 5′-TCCATCCTGCGAGCTCCTTG-3′ (F) and 5′-GCTGCATGGCACCTCTGTTC-3′ (R) for hsa_circ_0024353; 5′- GACTTGCAGGCTACGTTGAAGC-3′ (F) and 5′- CAAGTCCAGCTAGATCTGACACAAGAT-3′ (R) for hsa_circ_0085494; 5′- TCCTTTCTTGGCAACTGGAGGT-3′ (F), 5′-ACAAGTGAGGACAGCACGCA-3′ (R) for hsa_circ_0031408; and 5′-AGCCTCAAGATCATCAGCAATG-3′ (F) and 5′-ATGGACTGTGGTCATGAGTCCTT-3′ (R) for GAPDH.

### Prediction of miRNA Response Elements (MREs) and miRNA Target Genes

We used the CircInteractome (Dudekula et al., 2016) database to predict MREs of circRNAs. Target genes of predicted miRNAs were predicted by miRDB (Chen and Wang, 2020)^1^, miRTarBase (Chou et al., 2018)^2^, and TargetScan (Agarwal et al., 2015)^3^ databases.

### Construction of the CircRNA–miRNA–mRNA Interaction Network

We established a circRNA–miRNA–mRNA network to demonstrate the interactions occurring between mRNAs and circRNAs. The circRNA–miRNA potential correlation was predicted by the CircInteractome database, while miRNA-binding mRNAs were predicted by miRDB, miRTarBase, and TargetScan databases. To better understand key circRNAs, the circRNA–miRNA–mRNA interaction network was constructed using Cytoscape (Shannon et al., 2003) (version 3.6.1)^4^. The role of hub circRNAs was presented by Cytoscape.

### GO and KEGG Analyses

Gene Ontology (GO) and Kyoto Encyclopedia of Genes and Genomes (KEGG) analyses including gene set analyses were performed using the ConsensusPathDB-human (Kamburov et al., 2013)^5^ database.

### Validation of Target Genes by TCGA Database

The original mRNA expression profile used in the present study was downloaded from the TCGA database, and included 499 PCa tissues and 52 healthy prostatic tissues. Genes that were differentially expressed between tissues were screened for their interaction with predicted miRNA target genes.

### Validation of Target Genes Expression by GEPIA and HPA Database

We used GEPIA (Tang et al., 2017)^6^ to validate the mRNA expression of target genes. Protein expression was validated by the HPA (Uhlen et al., 2017)^7^ database.

## Results

### Differential Expression of CircRNAs in Patients With PCa

**FIGURE 1:**
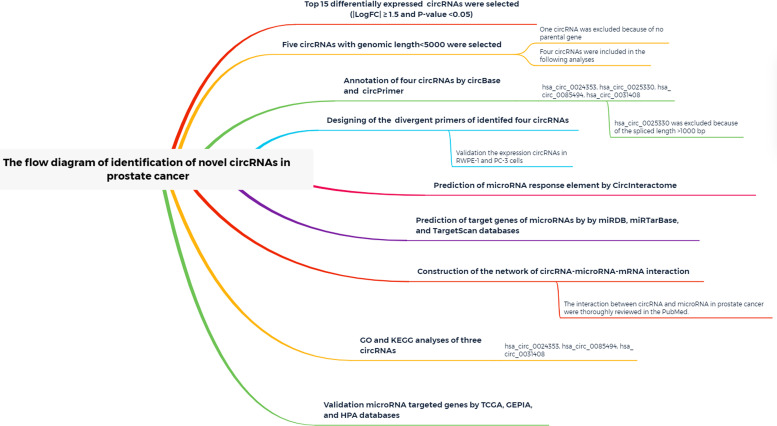
Study flow diagram.

A study flow diagram is presented in Figure 1. Expression profiles of the GSE140927 database (four PCa tissues and four paracancerous tissues of PCa) were screened, and GEO2R was used to identify differentially expressed circRNAs in patients with PCa. The top 250 RNAs, including 25 mRNAs, 165 lncRNAs, and 60 circRNAs, were obtained, and the 60 circRNAs were used for the following analysis.

### Selection of Screened CircRNAs

| LogFC| ≥ 1 and *P* < 0.05 were set as hub circRNA screened criteria. The top 15 circRNAs were identified as candidate circRNAs, including hsa_circ_0001407, hsa_circ_0050454, hsa_circ_0004529, hsa_circ_0024353, hsa_circ_0028880, hsa_circ_0044094, hsa_circ_0034546, hsa_circ_0045515, hsa_circ_0069230, hsa_circ_0025330, hsa_circ_0056631, hsa_circ_0082371, hsa_circ_0085494, hsa_circ_0069759, and hsa_circ_0031408. LogFC values of these circRNAs are shown in Table 1. We then used the circBase database to illustrate specific information about these circRNAs, including the position, strand, genomic length, spliced length, and gene symbol (Table 1). Only five circRNAs with genomic lengths less than 5 kb were included in this study: hsa_circ_0001407, hsa_circ_0024353, hsa_circ_0025330, hsa_circ_0085494, and hsa_circ_0031408. The corresponding gene symbol of each circRNA is shown in Table 1, and corresponding heatmaps are shown in Figure 2. No parental gene was found for hsa_circ_0001407, and hsa_circ_0025330 was excluded from further analysis because its spliced length exceeded 1 kb.

**TABLE 1:** Annotation of the top 15 differentially expressed circRNAs.

**ID**	**P.Value**	**logFC**	**Position**	**Strand**	**Genomic length**	**Spliced length**	**Gene symbol**
hsa_circ_0001407	0.000135	–2.28	chr4:42212390–42215180	+	2790	2790	None
hsa_circ_0050454	6.93E-05	–2.04	chr19:34287750–34306666	+	18916	4932	KCTD15
hsa_circ_0004529	0.000142	1.976	chr1:240286478–240351562	+	65084	371	FMN2
hsa_circ_0024353	0.000402	–1.96	chr11:116700623–116703787	+	3164	533	APOC3
hsa_circ_0028880	5.28E-05	–1.87	chr12:120884240–120894982	+	10742	499	GATC
hsa_circ_0044094	0.000208	–1.77	chr17:42875815–42908179	−	32364	7640	GJC1
hsa_circ_0034546	6.16E-05	1.55	chr15:39909935–40030402	−	120467	919	FSIP1
hsa_circ_0045515	0.000108	1.532	chr17:67080572–67109878	−	29306	2478	ABCA6
hsa_circ_0069230	0.000431	1.482	chr4:15707137–15724557	+	17420	663	BST1
hsa_circ_0025330	0.000488	1.477	chr12:7174946–7178335	+	3389	1381	C1S
hsa_circ_0056631	0.000475	–1.46	chr2:141272221–141299540	−	27319	1075	LRP1B
hsa_circ_0082371	0.000162	1.458	chr7:129940655–129964020	+	23365	2485	CPA4
hsa_circ_0085494	0.000357	1.438	chr8:125332326–125335616	−	3290	204	TMEM65
hsa_circ_0069759	0.000121	1.426	chr4:55146482–55151653	+	5171	283	PDGFRA
hsa_circ_0031408	0.000231	–1.41	chr14:25443870–25444056	−	186	186	STXBP6

**FIGURE 2:**
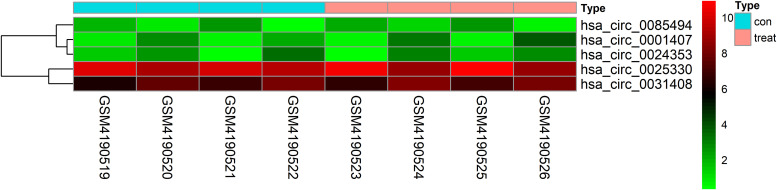
Heatmap of selected circRNAs. A total of five circRNAs were selected from four prostate cancer samples and four normal adjacent prostatic tissues samples. Red represents upregulation and green represents downregulation.

### Annotation of Hub CircRNAs

**FIGURE 3:**
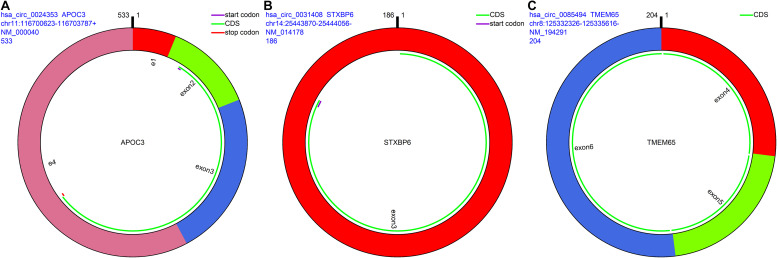
CircRNA annotation. The start codon, coding sequence, stop codon, parental gene symbol, position, and length are all presented in pattern graphs. **(A)** hsa_circ_0024353 is formed by the circularization of exon 2 and exon 3 of the *APOC3* gene. **(B)** hsa_circ_0031408 is formed by the circularization of exon 3 of the *STXBP6* gene. **(C)** hsa_circ_0085494 is formed by the circularization of exons 4–6 of the *TMEM65* gene.

**FIGURE 4:**
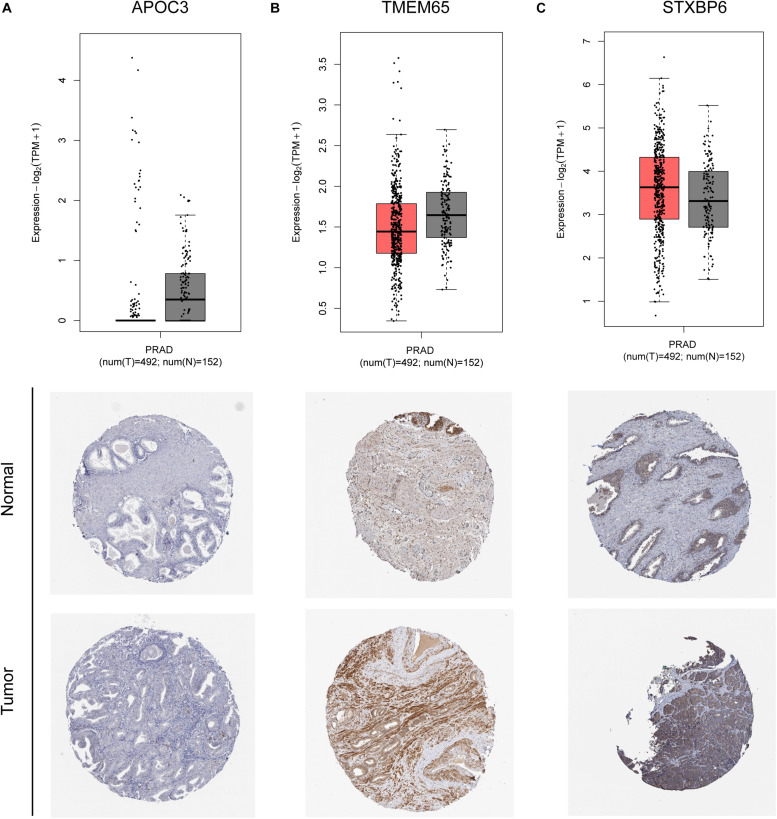
mRNA and protein expression of *APOC3* **(A)**, *TMEM65* **(B)**, and *STXBP6* **(C)** genes of identified circRNAs.

The remaining three circRNAs (hsa_circ_0024353, hsa_circ_0085494, hsa_circ_0031408) were annotated by circPrimer. We next used circBase and circPrimer software to annotate these circRNAs. Corresponding gene symbols are *APOC3* for hsa_circ_0024353 (Figure 3A), *TMEM65* for hsa_circ_0085494 (Figure 3B), and *STXBP6* for hsa_circ_0031408 (Figure 3C). hsa_circ_0024353 is formed by the circularization of exon 2 and exon 3 of the *APOC3* gene, hsa_circ_0031408 is formed by the circularization of exon 3 of the *STXBP6* gene, and hsa_circ_0085494 is formed by the circularization of exons 4–6 of the *TMEM65* gene. mRNA and protein expression levels of these corresponding genes are shown in Figures 4A–C.

### Validation of the Expression of Identified CircRNAs in PCa

**FIGURE 5:**
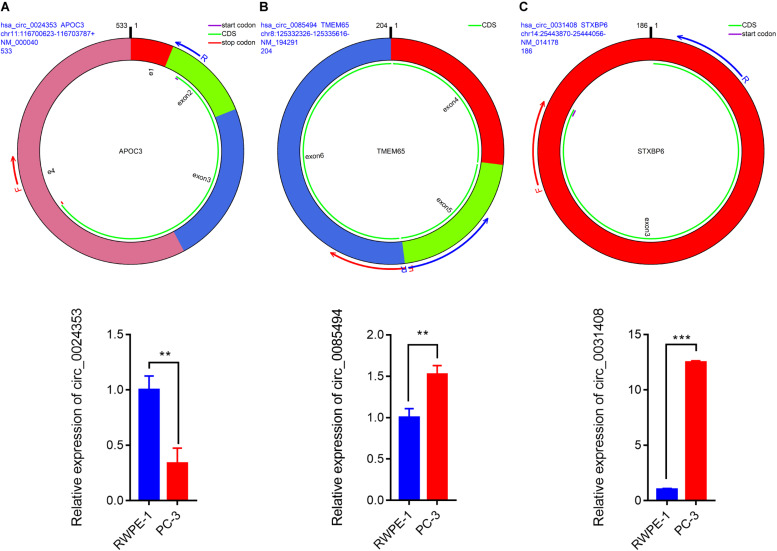
Pattern diagrams of divergent primers spanning back-splicing junctions and qRT-PCR validation of the expression of hsa_circ_0024353 **(A)**, hsa_circ_0085494 **(B)**, and hsa_circ_0031409 **(C)** in RWPE-1 and PC-3 cells. *P < 0.05, **P < 0.01, and ***P < 0.001.

We used circPrimer (version 1.2) and Primer3 (version 4.1) software to design divergent primers for hub circRNAs. Primer diagrams are shown in Figures 5A–C. qRT-PCR was performed to validate the expression of identified circRNAs in RWPE-1 and PC-3 cells. The results of qRT-PCR demonstrated that the expression of hsa_circ_0024353 was significantly downregulated in PC-3 cells when compared with RWPE-1 cells, while the expression of hsa_circ_0031408 and hsa_circ_0085494 was significantly upregulated in PC-3 cells when compared with RWPE-1 cells (Figures 5A–C).

### MRE Prediction

The CircInteractome database predicted a total of 26, 10, and eight MREs for hsa_circ_0024353, hsa_circ_0031408, and hsa_circ_0085494, respectively (Table 2).

**TABLE 2:** Predicted miRNA response elements of circRNAs.

**CircRNA**	**MicroRNA response element**
hsa_circ_0024353	hsa-miR-1234, hsa-miR-1250, hsa-miR-1253, hsa-miR-127-5p, hsa-miR-1286, hsa-miR-183, hsa-miR-198, hsa-miR-324-5p, hsa-miR-331-3p, hsa-miR-338-3p, hsa-miR-339-3p, hsa-miR-370, hsa-miR-486-3p, hsa-miR-503, hsa-miR-578, hsa-miR-580, hsa-miR-605, hsa-miR-1270, hsa-miR-620, hsa-miR-646, hsa-miR-661, hsa-miR-665, hsa-miR-767-3p, hsa-miR-940, hsa-miR-941
hsa_circ_0031408	hsa-miR-145, hsa-miR-182, hsa-miR-192, hsa-miR-215, hsa-miR-335, hsa-miR-338-3p, hsa-miR-502-5p, hsa-miR-513a-3p, hsa-miR-890
hsa_circ_0085494	hsa-miR-203, hsa-miR-330-3p, hsa-miR-619, hsa-miR-646, hsa-miR-649, hsa-miR-671-5p, hsa-miR-873, hsa-miR-95

### Prediction of miRNA Target Genes

Target genes of predicted miRNAs were predicted by miRDB, miRTarBase, and TargetScan databases, and interactions among these three databases were obtained. The selected genes were used in the construction of the circRNA–miRNA–mRNA interaction network.

### Construction of the CircRNA–microRNA–mRNA Interaction Network

**FIGURE 6:**
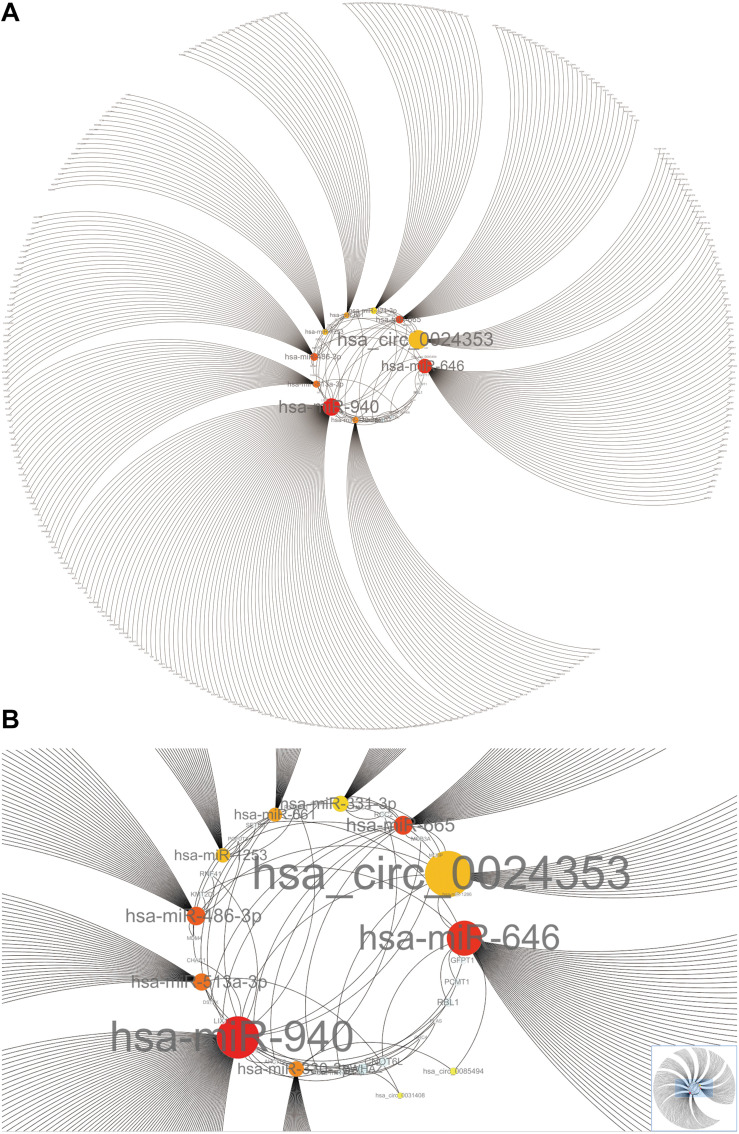
The circRNA–miRNA-mRNA interaction network. **(A)** Overview of the entire network. **(B)** Specific relationship between circRNAs and interacting miRNAs in the network. Sizes of characters and circles represent the weight of circRNAs and miRNAs.

Hub circRNAs and mRNAs contain corresponding miRNA binding sites. Interactions between circRNAs and miRNAs were predicted by the Circular RNA Interactome online tool and combined with interactions identified between miRNAs and corresponding target genes. The circRNA–microRNA–mRNA interaction network was constructed using Cytoscape (version 3.6.1) software, and the roles of hub circRNAs are shown in Figures 6A,B. Previous publications describing interactions between circRNAs and miRNAs in PCa were thoroughly reviewed in the PubMed database. A total of 13 studies were identified describing 18 circRNA–miRNA interactions (Table 3).

**TABLE 3:** PubMed literature review of circRNA–miRNA interactions.

**Authors**	**circRNA**	**microRNA**	**Gene**
Zheng et al.	circ_KATNAL1	miR-145-3p	WISP1
Song et al.	circ_0001206	miR-1285-5p	Smad4
Zheng et al.	circ_SLC19A1	miR-497	Septin 2
Xia et al.	circ_0057558	miR-6884	
	circ_0034467	miR-6884	
	circ_0062019	miR-5008	
	circ_0060325	miR-5008	
Tian et al.	circ_0044516	miR-29a-3p	
Yan et al.	circ_0001165	miR-187-3p	TNF
	circ_0001085	miR-196b-5p	
	circ_0001085	miR-451a	
Wang et al.	circITCH	miR-17-5p	HOXB13
Dai et al.	circ_0141940	miR-29a	
Hu et al.	circ-MTO1	miR-17-5p	
Wu et al.	circ_0001427	miR-181c-5p	ARv7
Qu et al.	circAMOTL1	miR-485-5p	AMOTL1
Kong et al.	circFOXO3	miR-29a-3p	SLC25A15
Shan et al.	circFMN2	miR-1238	LHX2

### GO and KEGG Analyses

GO and KEGG analyses of potential target genes of hsa_circ_0024353, hsa_circ_0031408, and hsa_circ_0085494 were performed by ConsensusPathDB.

#### hsa_circ_0024353

**FIGURE 7:**
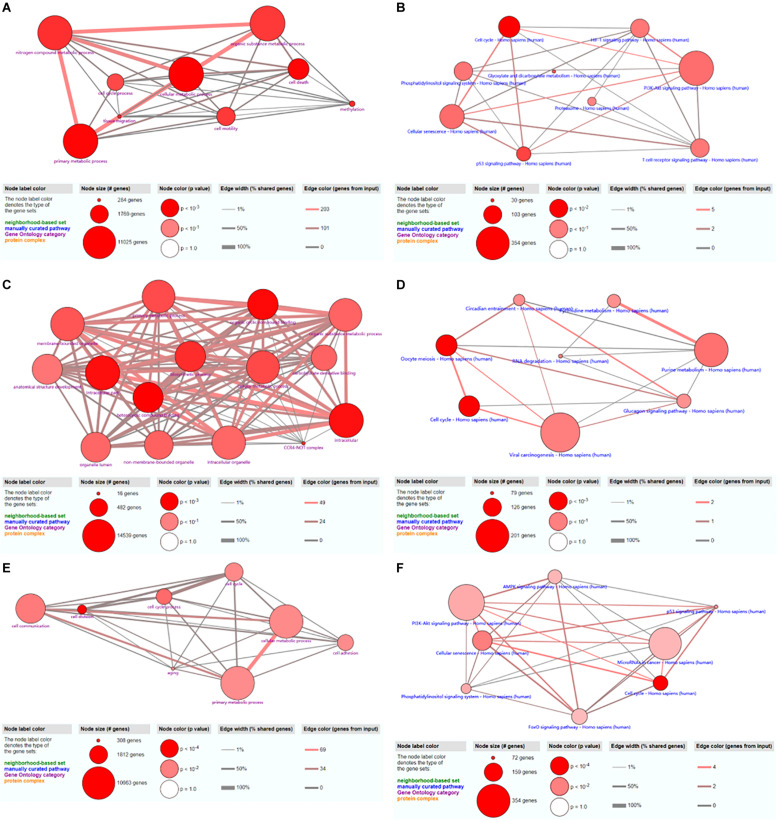
GO and KEGG analyses of hsa_circ_0024353 **(A,B)**, hsa_circ_0031408 **(C,D)**, and hsa_circ_0085494 **(E,F)**.

A total of 48 GO terms were shown to be enriched by ConsensusPathDB. These potential target genes were mainly enriched in the primary metabolic process, cellular metabolic process, organic substance metabolic process, and nitrogen compound metabolic process (Figure 7A). A total of 26 pathways were shown to be enriched by ConsensusPathDB. These potential target genes were mainly enriched in the phosphoinositide 3-kinase (PI3K)-Akt signaling pathway, cellular senescence, the cell cycle, the hypoxia-inducible factor (HIF)-1 signaling pathway, the T cell receptor signaling pathway, the p53 signaling pathway, and the phosphatidylinositol signaling system (Figure 7B).

#### hsa_circ_0031408

A total of 15 GO terms were shown to be enriched by ConsensusPathDB. These potential target genes were mainly enriched in the primary metabolic process, cellular metabolic process, organic substance metabolic process, and the intracellular process (Figure 7C). A total of 16 pathways were shown to be enriched by ConsensusPathDB. These potential target genes were mainly enriched in viral carcinogenesis, purine metabolism, oocyte meiosis, and the cell cycle (Figure 7D).

#### hsa_circ_0085494

A total of 40 GO terms were shown to be enriched by ConsensusPathDB. These potential target genes were mainly enriched in cell communication, cellular metabolic process, and primary metabolic process (Figure 7E). A total of 17 pathways were shown to be enriched by ConsensusPathDB. These potential target genes were mainly enriched in the PI3K-Akt signaling pathway, miRNAs in cancer, and the AMP-activated protein kinase (AMPK) signaling pathway (Figure 7F).

### Validation of miRNA Target Genes by TCGA Database

**FIGURE 8:**
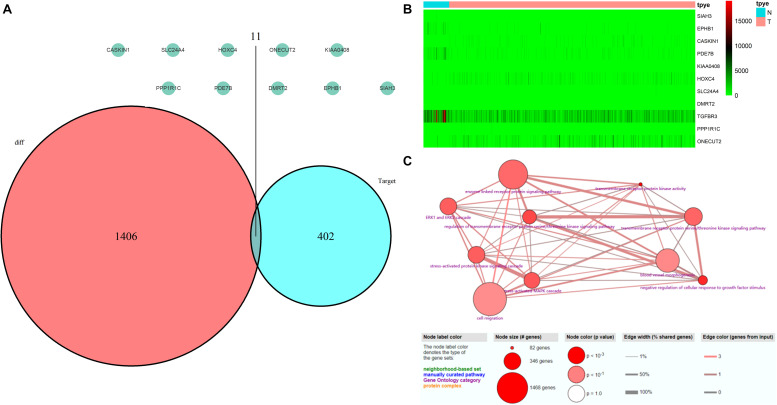
Validation of miRNA-regulated genes by the TCGA database. **(A)** Interaction between predicted miRNA-regulated genes and differentially expressed genes between PCa and normal adjacent prostatic tissues selected from the TCGA database. **(B)** Heatmap generated from data of 552 samples downloaded from the TCGA database. **(C)** GO terms analyses of these 11 genes.

A total of 552 samples were downloaded from the TCGA database, including 52 normal adjacent tissues and 499 PCa samples. The clinicopathological characteristics of 367 patients with PCa from TCGA cohort were demonstrated in Table 4 (Patients with incomplete clinicopathological data were excluded). A comparison of these was used to identify a total of 1417 differentially expressed genes (| fold change| > 1.5 and *P* < 0.05). A total of 413 predicted differentially expressed mRNAs were also identified. Venn diagrams were used to select 11 overlapping genes, including *SIAH3*, *EPHB1*, *CASKIN1*, *PDE7B*, *KIAA0408*, *HOXC4*, *SLC24A4*, *DMRT2*, *TGFBR3*, *PPP1R1C*, and *ONECUT2* (Figure 8A). The heatmap of these 11 genes was generated based on data from 552 samples downloaded from the TCGA database (Figure 8B). GO analyses of these 11 genes were also performed (Figure 8C).

**TABLE 4:** Clinicopathological characteristics of 367 patients with PCa from TCGA cohort.

**Clinicopathological characteristics**	**Value**
**Age, y**	
Mean ± SD	61.37 ± 6.68
Range	41–78
**T stage, n (%)**	
T2	117 (31.9)
T3	238 (64.8)
T4	8 (2.2)
Unknown	4 (1.1)
**N stage, n (%)**	
N0	291 (79.3)
N1	76 (20.7)

### Validation of Expression in PCa Tissues by GEPIA and HPA Databases

**FIGURE 9:**
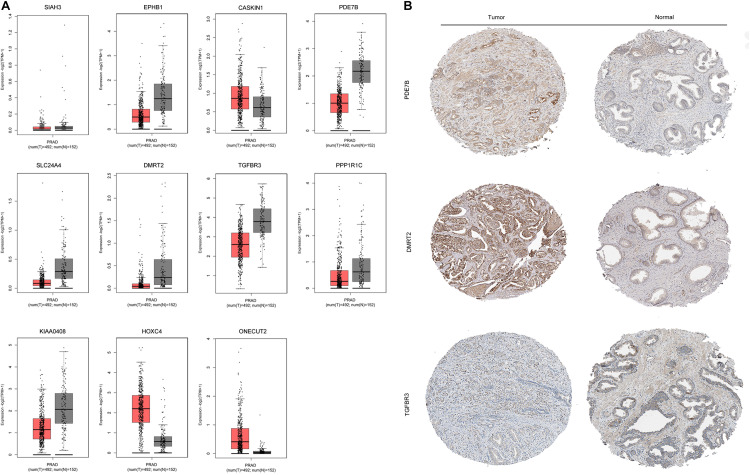
Validation of miRNA-regulated genes by GEPIA and HPA databases. **(A)** Differentially expressed genes identified in the GEPIA database. **(B)** Differentially expressed genes identified in the HPA database.

The mRNA expression of the 11 differentially expressed genes was validated in the GEPIA database (Figure 9A). *CASKIN1*, *HOXC4*, and *ONECUT2* were found to be upregulated in PCa compared with normal prostatic tissues. However, *EPHB1*, *PDE7B*, *SLC24A4, DMRT2*, *TGFBR3*, *PPP1R1C*, and *KIAA0408* were downregulated in PCa tissues compared with normal prostatic tissues. *SIAH3* showed no difference in expression between PCa tissues and normal prostatic tissues. To investigate the expression of proteins encoded by these genes, immunohistochemical assays performed in the HPA database were analyzed (Figure 9B). PDE7B and DMRT2 protein expression was found to be higher in PCa tissues than in normal prostatic tissues, which is inconsistent with mRNA findings from the GEPIA database. However, TGFBR3 protein expression was shown to be lower in PCa tissues than in normal prostatic tissues, which is consistent with GEPIA database results.

## Discussion

CircRNAs are widely reported to be associated with a range of biological processes and to participate in many types of cancers (Meng et al., 2017; Bi et al., 2018). They have multiple functions, including acting as scaffolds in the assembly of protein complexes, modulating the expression of parental genes, and regulating RNA–protein interactions. One of their most important roles is functioning as miRNA sponges (Hansen et al., 2013). These are defined as competitive inhibitors of small RNAs in mammalian cells (Ebert et al., 2007), and circRNAs miRNA sponges regulate gene expression through their interactions (Han et al., 2017). Although previous studies (Chen et al., 2017; Meng et al., 2017; Kristensen et al., 2018) have reported circRNA miRNA sponge functions in many types of cancers, little is known about the role of circRNAs in PCa.

Kong et al. (2020) showed that hsa_circ_0006404 promoted PCa progression through sponging miR-29a-3p, while Cai et al. (2019) demonstrated that circHIPK3 overexpression accelerated the proliferation and invasion of PCa cells by regulating miRNA-339-3p. Additionally, Wang et al. (2019) revealed that circRNA ITCH suppressed PCa progression by upregulating HOXB13 expression through sponging miR-17-5p. In the present study, we used bioinformatics analyses to identify circRNAs that were differentially expressed between PCa tissues and corresponding normal adjacent prostatic tissues.

We screened and identified the top 250 RNAs from GSE140927 expression profiles, including 60 circRNAs. Of the top 15 circRNAs, only those with a genomic length less than 5 kb, a spliced length < 1 kb, and an identified parental gene symbol were selected for further analysis (hsa_circ_0024353, hsa_circ_0085494, and hsa_circ_0031408). The roles of these circRNAs in cancer, especially PCa, have not been explored.

CircRNAs have previously been reported to regulate gene expression by MREs (Hansen et al., 2013). We therefore used the CircInteractome database to predict 26, 10, and eight MREs for hsa_circ_0024353, hsa_circ_0031408, and hsa_circ_0085494, respectively, and used these to predict target genes by miRDB, miRTarBase, and TargetScan databases. GO and KEGG analyses showed that the predicted genes were mainly enriched in metabolic-related processes. Interactions among these databases enabled 11 genes to be selected which were mainly enriched in cell migration and enzyme-linked receptor protein signaling pathways.

We used Cytoscape to construct a circRNA–miRNA–mRNA interaction network which revealed that hsa_circ_0024353 interacts with the most dominant miRNAs such as hsa-miR-646, hsa-miR-940, hsa-miR-665, and hsa-miR-1253. Following GEPIA and HPA database validation of mRNA and protein expression of the 11 genes, *PDE7B*, *DMRT2*, and *TGFBR3* were identified as novel genes regulated by the identified circRNAs and with potential roles in PCa. *PDE7B* is the target gene of hsa-miR-940 which can be regulated by hsa_circ_0024353; *DMRT2* is the target gene of hsa-miR-1253 which can also be regulated by hsa_circ_0024353; and *TGFBR3* is the target gene of hsa-miR-330-3p which can be regulated by hsa_circ_0085494.

The circRNAs identified in this study have not been reported previously. Therefore, we performed a literature review of circRNAs in PCa in the PubMed database, identifying 13 studies (Xia et al., 2018; Shen et al., 2019; Song et al., 2019; Wang et al., 2019; Wu et al., 2019; Hu and Guo, 2020; Kong et al., 2020; Li T. et al., 2020; Ou et al., 2020; Shan et al., 2020; Yan et al., 2020; Zheng et al., 2020a, b) of 18 circRNA–miRNA interactions. The miRNAs identified in this study have previously been shown to play important roles in PCa. Chen et al. (2018) found that long-non-coding (lnc)RNA GAS5 and ZFAS1 can be used as prognostic markers in PCa and that they are involved in translation targeted by miR-940 in PCa, while lncRNA FOXC2-AS1 was reported to promote proliferation and progression in PCa by targeting miR-1253/EZH2 (Chen et al., 2019). Liu et al. (2019) demonstrated that lncRNA LEF1-AS1 knockdown suppressed the initiation and development of PCa by targeting miR-330-5p. *PDE7B*, *DMRT2*, and *TGFBR3* are involved in several types of tumor such as lung cancer (Liu et al., 2018), bladder cancer (Liu et al., 2013), and head and neck cancer (Fang et al., 2020). However, their roles in PCa have not been fully clarified. de Alexandre et al. (2015) suggested that *PDE7B* is involved in the regulation of cAMP and cyclic guanosine monophosphate levels associated with PCa. Nishan et al. (2019) demonstrated a role for *DMRT2* in PCa patients after castration, while Sharifi et al. (2007) found that the downregulation of *TGFBR3* is an important step in prostate tumorigenesis. Our findings can be used to predict three circRNA–miRNA–mRNA interaction axes that appear to be involved in PCa: hsa_circ_0024353–hsa-miR-940–PDE7B, hsa_circ_0024353–hsa-miR-1253–DMRT2, and hsa_circ_0085494–hsa-miR-330-3p–TGFBR3.

## Data Availability Statement

The datasets presented in this study can be found in online repositories. The names of the repository/repositories and accession number(s) can be found in the article/supplementary material.

## Author Contributions

Y-PW: conceptualization. X-DL, Z-BK, and S-HC: data curation. FL: formal analysis. X-YX and D-NC: investigation. Y-PW, NX, and YW: methodology. YW, Q-SZ, and X-YX: project administration. Q-SZ: visualization. Y-PW, Z-BK, X-DL, and S-HC: writing – original draft. Y-AW and NX: writing – review and editing. All authors contributed to the article and approved the submitted version.

## Conflict of Interest

The authors declare that the research was conducted in the absence of any commercial or financial relationships that could be construed as a potential conflict of interest.

## Footnotes

1 http://mirdb.org/

2 http://mirtarbase.mbctu.edu.tw/php/index.php

3 http://www.targetscan.org/vert_72/

4 https://cytoscape.org/

5 http://cpdb.molgen.mpg.de/

6 http://gepia.cancer-pku.cn/

7 https://www.proteinatlas.org/

## Abbreviations

ADT: androgen deprivation therapy
CDS: the coding sequence
circRNAs: circular RNAs
GEO: Gene Expression Omnibus
GO: Gene ontology
KEGG: Kyoto Encyclopedia of Genes and Genomes
MRE: microRNA response element
PCa: Prostate cancer
UTR: untranslated region.
